# mTORC1 phosphorylates and stabilizes LST2 to negatively regulate EGFR

**DOI:** 10.1073/pnas.2405959121

**Published:** 2024-08-14

**Authors:** Stefania Battaglioni, Louise-Marie Craigie, Sofia Filippini, Timm Maier, Michael N. Hall

**Affiliations:** ^a^Biozentrum, University of Basel, Basel 4056, Switzerland

**Keywords:** mTOR signaling, TOS motif, phosphorylation substrate, negative feedback, LST2

## Abstract

TORC1 (target of rapamycin complex 1) is a highly conserved protein kinase that plays a central role in regulating cell growth. Given the role of mammalian TORC1 (mTORC1) in metabolism and disease, understanding mTORC1 downstream signaling and feedback loops is important. mTORC1 recognizes some of its substrates via a five amino acid binding sequence called the TOR signaling (TOS) motif. mTORC1 binding to a TOS motif facilitates phosphorylation of a distinct, distal site. Here, we show that LST2, also known as ZFYVE28, contains a TOS motif (amino acids 401 to 405) and is directly phosphorylated by mTORC1 at serine 670 (S670). mTORC1-mediated S670 phosphorylation promotes LST2 monoubiquitination on lysine 87 (K87). Monoubiquitinated LST2 is stable and displays a broad reticular distribution. When mTORC1 is inactive, unphosphorylated LST2 is degraded by the proteasome. The absence of LST2 enhances EGFR (epidermal growth factor receptor) signaling. We propose that mTORC1 negatively feeds back on its upstream receptor EGFR via LST2.

## Significance

Dysregulation of mTORC1 signaling has been linked to major disease, yet relatively few mTORC1 phosphorylation substrates have been identified. We show that LST2 is a mTORC1 substrate. mTORC1, through LST2 phosphorylation, generates a feedback loop to inhibit its upstream activator EGFR. These findings provide insight into the regulation of the mTORC1 signaling pathway and may impact therapeutic strategies.

Mechanistic target of rapamycin (mTOR, also known as mammalian TOR) is an essential serine/threonine protein kinase that responds to nutrients, growth factors, and cellular energy to promote cell growth. Due to mTOR’s central role in controlling cell growth and metabolism, mTOR signaling dysregulation is implicated in multiple diseases, including cancer, diabetes, and neurological disorders (1, 2). However, despite over 30 y of research, a comprehensive understanding of mTOR substrates and their function is still lacking (3). Elucidating the full extent of mTOR signaling will provide crucial insight to understand, and potentially treat, disease.

mTOR nucleates two distinct complexes, mTORC1 and mTORC2, each characterized by specific subunits, targets, downstream signaling pathways, and functions (4, 5). mTORC1 contains mTOR, regulatory-associated protein of mTOR (RAPTOR), and mammalian lethal with SEC13 protein 8 (mLST8) (4, 6, 7). RAPTOR binds mTORC1 substrates, including the well-characterized substrates ribosomal protein S6 kinase 1 (S6K1) and 4E binding protein 1 (4E-BP1), through a five-amino acid motif termed TOS. RAPTOR binding to the TOS motif facilitates the presentation of the target phosphorylation site to the mTOR kinase catalytic pocket. The consensus TOS motif is F-X-Φ-(E/D)-Φ, where Φ represents a hydrophobic residue and X is any residue (3, 8, 9). Mutation of the invariant phenylalanine (F) is sufficient to abrogate RAPTOR binding (9). Although only few of the known mTORC1 substrates contain a TOS motif, TOS binding is the best-characterized mode of mTORC1 substrate recognition (3). Here, we report the characterization of a TOS motif in lateral signal target 2 homolog (LST2) protein. We also show that LST2 is an mTORC1 substrate and part of a negative feedback loop in mTORC1 signaling.

LST2 is a poorly characterized protein. It contains a FYVE domain, which typically binds to phosphatidylinositol 3-phosphate (PI3P) on early endosomes (10), but GFP-tagged LST2 shows a reticular rather than endosomal distribution (11). However, LST2 is constitutively monoubiquitinated on K87 and mutation of this site confers endosomal localization (11). Although ubiquitination is generally a modification targeting a protein for proteasomal degradation, it can also perform nonproteolytic functions. Indeed, ubiquitination can mediate protein stabilization, localization, or activity (12, 13).

LST2 has been proposed to negatively regulate the receptor tyrosine kinases EGFR and insulin receptor (11, 14–16). EGFR promotes proliferation and survival in epithelial cells. EGFR is a common oncoprotein, in lung, breast, brain, and colorectal cancer, where EGF signaling is hyperactive (17). EGFR is present in the plasma membrane in an autoinhibited state but once bound by epidermal growth factor (EGF) undergoes dimerization and autophosphorylation that triggers the activation of several downstream signaling cascades, including the PI3K–AKT–mTOR and MAPK/ERK pathways. Ligand-bound EGFR is internalized by the endosomal pathway to be either recycled back to the plasma membrane or degraded in the lysosome. The balance between these two fates is crucial in achieving signaling homeostasis. LST2 is proposed to interfere with EGFR recycling and to promote EGFR degradation (11). Defects in EGFR recycling or degradation have been linked to hyperactive EGFR signaling and cancer (18).

Here, we describe a negative feedback loop between mTORC1 and EGFR. We show that LST2, as first suggested by a putative TOS motif, is indeed an mTORC1 substrate. Furthermore, we show that mTORC1 phosphorylates LST2 to dampen EGFR signaling.

## Results

### The LST2 TOS Peptide Binds RAPTOR.

**Fig. 1.**
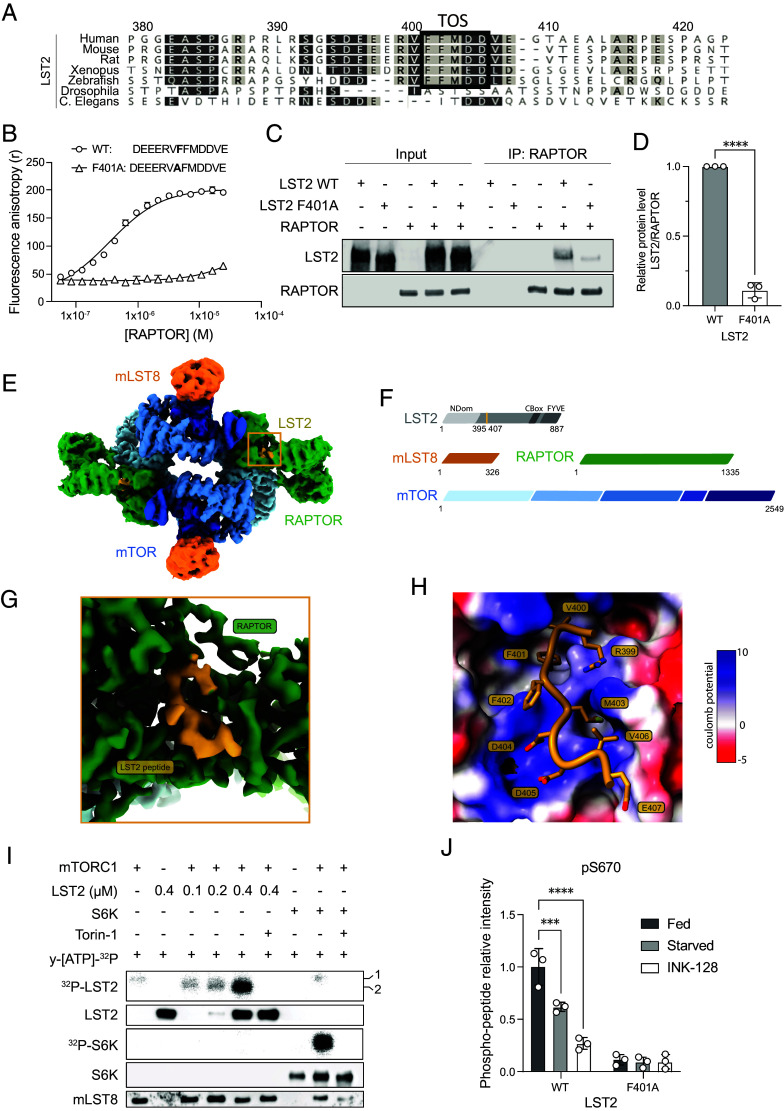
LST2 binds RAPTOR through its TOS motif and is phosphorylated by mTORC1 on S670. (*A*) Sequence alignment of the LST2 protein from different species. Uniprot ID of human: Q9HCC9, mouse: Q6ZPK7, rat: A0A8I5ZMR3, *Xenopus*: Q0P4S0, zebrafish: A0JMD2, drosophila: Q9VB70 and C. elegans: Q9TZD0. The TOS motif, which is conserved in vertebrates, is indicated by a black box. (*B*) Binding of synthetic N-terminally FITC labeled peptides representing the WT and F401A mutant of the LST2 TOS motif to RAPTOR measured by fluorescence anisotropy. Kd (WT) = 330 nM, N = 3. (*C*) Coimmunoprecipitation of full-length LST2-WT or -F401A mutant with RAPTOR. LST2 is 96 kDa and runs at 130 kDa on SDS-PAGE, as previously shown in (11). (*D*) Quantification of C. Unpaired t test, N = 3, ****P < 0.0001. (*E*) Cryo-EM reconstruction of mTORC1 in the presence of full-length LST2. A composite map from two locally refined protomers (map 1 from *SI Appendix*, Fig. S2) is contoured at 10.5 electron/Å^2^. Map regions are colored according to proteins, LST2 is colored yellow and indicated by a box, RAPTOR in green, mLST8 in orange, and mTOR in shades of blue, as in panel *F*. (*F*) Schematic representation of the domain architecture of mTORC1 and LST2. The resolved region of LST2 is indicated in yellow. (*G*) Cryo-EM reconstruction of the LST2 TOS peptide bound to mTORC1. A map at 3.27 Å resolution (map 6 from *SI Appendix*, Fig. S3) from a local refinement is contoured at 0.52 electron/Å^2^, displaying the same region indicated in panel E by a box, coloring corresponds to panel *E*, *F*. (*H*) Interactions of the LST2 TOS peptide bound to mTORC1. Surface representation of the RAPTOR TOS-binding pocket region colored by charge (blue and red indicating positive and negative charge, respectively). The LST2 peptide is shown in cartoon representation in yellow with side chains displayed in stick representation with dark yellow for carbon atoms, light yellow for sulfur, blue for nitrogen, and red for oxygen. LST2 peptide amino acids are labeled. (*I*) Radioactive in vitro mTORC1 kinase assay. Each reaction was carried out with 10 µM radioactive [γ-32P]-ATP (2.5 µCi). The SDS-PAGE gel was first exposed for radioactive detection then transferred and membrane probed with indicated antibodies. (*J*) Quantitative mass spectrometric analysis of LST2 phosphorylation on S670. Intensities have been normalized for WT Fed condition. Two-way ANOVA, N = 3, ***P < 0.001, ****P < 0.0001.

To determine whether a putative TOS motif in LST2 is functionally significant, we first performed a sequence alignment of LST2 from various species. The LST2 protein is conserved from worms to human, but the LST2 TOS sequence is found only in vertebrates (Fig. 1*A*). To assess whether the LST2 TOS motif is functional, we examined whether a synthetic peptide corresponding to the LST2 TOS sequence binds RAPTOR. We performed fluorescence anisotropy with purified RAPTOR protein and a FITC-labeled LST2 TOS peptide including flanking residues (amino acids 395 to 407) (Fig. 1*B*). The TOS peptide bound RAPTOR with a dissociation constant (Kd) of 330 nM. Furthermore, unlabeled TOS peptide competed with FITC-labeled peptide for RAPTOR binding (*SI Appendix,* Fig. S1*A*). The TOS–RAPTOR interaction was abrogated by a single residue replacement in the TOS peptide equivalent to a F401A point mutation (Fig. 1*B*). Next, we performed a coimmunoprecipitation experiment with purified RAPTOR and full-length LST2 (WT and F401A), to determine whether the TOS motif in full-length LST2 is exposed and accessible for binding. RAPTOR clearly bound LST2-WT whereas the binding of RAPTOR to LST2-F401A was reduced 9-fold (Fig. 1 *C* and *D*). Thus, RAPTOR binds the LST2 TOS sequence with sub-micromolar affinity and LST2 F401 is critical for binding.

To characterize the binding of LST2 to mTORC1, we performed cryo-EM single-particle analysis on mTORC1 in complex with full-length LST2. An overall refinement of mTORC1 in complex with LST2 was obtained at 4.02 Å resolution (*SI Appendix,* Fig. S2 and Table S1). Interestingly, density representing LST2 was observed only in the RAPTOR TOS-binding pocket, suggesting that mTORC1 recognizes LST2 solely through the TOS sequence (Fig. 1 *E* and *F* and *SI Appendix*, Fig. S1*B*). To further improve the resolution of the visualization of TOS motif interactions, we analyzed mTORC1 in complex with the synthetic LST2 TOS peptide, for which higher concentrations could be reached compared to full-length LST2. Single-particle cryo-EM analysis achieved a resolution of 3.27 Å (map6—*SI Appendix,* Fig. S3 and Table S1) for the TOS-binding RAPTOR region. This allowed atomic modeling of nine peptide residues (corresponding to amino acids 399 to 407 in full-length LST2), including the TOS motif, in the RAPTOR TOS-binding pocket (Fig. 1 *G* and *H*). The LST2 TOS peptide contains three negatively charged residues which make electrostatic interactions with the positively charged TOS-binding pocket. F401 and M403 anchor the TOS peptide by binding in particularly deep cavities in the TOS-binding site, via hydrophobic interactions with surrounding residues. Finally, a comparison of LST2 TOS and previously described 4E-BP1 TOS binding shows that the TOS peptides, in particular, the consensus phenylalanine (F401 in LST2 and F114 in 4E-BP1), share a common mode of binding to the RAPTOR pocket (*SI Appendix,* Fig. S1*D*) (9). Overall, LST2 binds exclusively via its TOS sequence to mTORC1 in a canonical RAPTOR–TOS interaction, as previously described for 4E-BP1 and other TOS sequences (9, 19).

### mTORC1 Phosphorylates LST2 S670.

To determine whether LST2 is an mTORC1 substrate, as suggested by the LST2 TOS sequence, we performed an in vitro kinase assay with purified mTORC1 and full-length LST2. mTORC1 efficiently phosphorylated wild-type LST2, but not LST2-F401A, in vitro (Fig. 1*I* and *SI Appendix*, Fig. S1 *E* and *F*), indicating that mTORC1 phosphorylates LST2 in a TOS-dependent manner. To identify the residues phosphorylated by mTORC1, we analyzed phosphorylated LST2 using two approaches. First, LST2 was phosphorylated in an in vitro kinase assay in the presence or absence of the mTOR inhibitor INK-128 (20). Second, LST2 was phosphorylated in vivo. Overexpressed LST2 (WT and F401A) was immunoprecipitated from cells serum-starved overnight and refed for 2 h with serum, with serum plus INK-128, or kept in serum-deprived media (starved). In both approaches, the LST2 protein was subjected to mass spectrometry to identify potential phosphorylation sites (Fig. 1*J* and *SI Appendix*, Fig. S1 *G* and *H* and Dataset S1). S670 was the most phosphorylated site and the only site common to both approaches, although other phospho-sites were also detected (S334 and S368, *SI Appendix,* Fig. S1 *G* and *H* and Dataset S1). S670 phosphorylation was reduced upon starvation or INK-128 treatment, indicating that this site was phosphorylated in an mTORC1-dependent manner (Fig. 1*J* and *SI Appendix*, Fig. S1 *G* and *H* and Dataset S1). Thus, LST2 is phosphorylated by mTORC1, primarily at S670.

### LST2 S670 Phosphorylation Is Necessary for LST2 Stabilization.

**Fig. 2.**
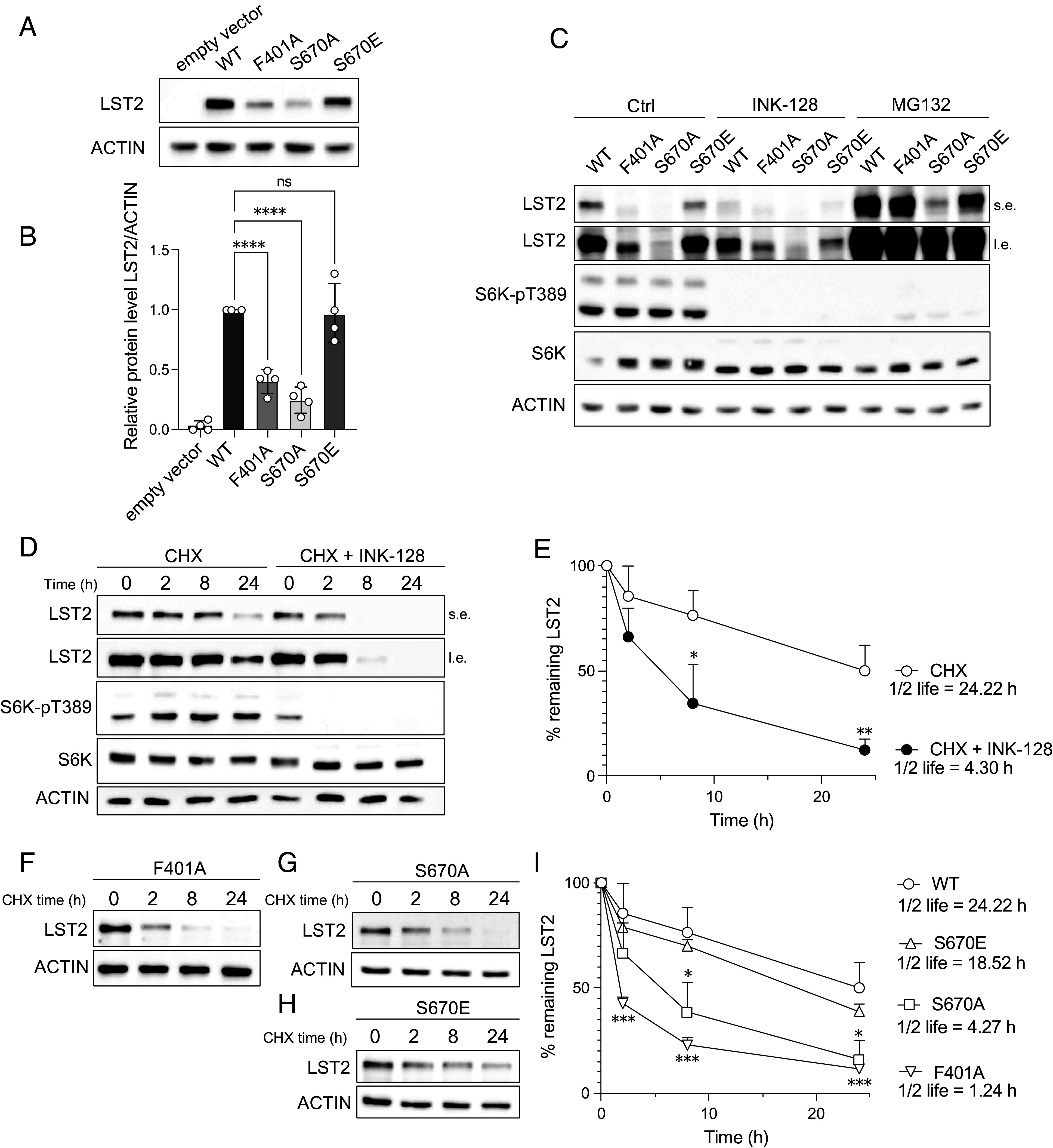
LST2 is stabilized by mTORC1-mediated phosphorylation. (*A*) Immunoblots upon empty vector and LST2-WT, LST2-F401A, LST2-S670A, LST2-S670E overexpression in HEK293T cells. Media replenished 4 h before cell collection. ACTIN serves as a loading control. (*B*) Quantification of A. One-way ANOVA, N = 4, ****P < 0.0001. (*C*) Immunoblots upon LST2-WT, LST2-F401A, LST2-S670A, LST2-S670E overexpression in HeLa cells. Cells were treated with DMSO (Ctrl), 200 nM INK-128, or 10 µM MG132 for 20 h. ACTIN serves as a loading control. (*D*) Immunoblots upon LST2-WT overexpression in HEK293T cells treated with 100 µg/mL cycloheximide (CHX) with or without 200 nM INK-128 for the indicated times. ACTIN serves as a loading control. (*E*) Quantification of D. Nonlinear fit. Inhibitor vs. response (three parameters), N = 3. Multiple unpaired t test, N = 3, *P < 0.05, **P < 0.01. Statistical difference is shown comparing CHX + INK-128 to CHX in the illustrated time points. (*F*) Immunoblots upon LST2-F401A overexpression in HEK293T cells treated with 100 µg/mL cycloheximide (CHX) for the indicated times. ACTIN serves as a loading control. (*G*) Immunoblots upon LST2-S670A overexpression in HEK293T cells treated with 100 µg/mL cycloheximide (CHX) for the indicated times. ACTIN serves as a loading control. (*H*) Immunoblots upon LST2-S670E overexpression in HEK293T cells treated with 100 µg/mL cycloheximide (CHX) for the indicated times. ACTIN serves as a loading control. (*I*) Quantification of D, F, G, and H. Nonlinear fit. Inhibitor vs. response (three parameters), N = 3. Multiple unpaired t test, N = 3, *P < 0.05, ***P < 0.001. Statistical difference is shown for the specific mutant in comparison to WT in the illustrated time points.

Next, we examined the functional relevance of mTORC1-mediated LST2 phosphorylation. Phospho-deficient (S670A) and phospho-mimetic LST2 mutants (S670E), in all cases N-terminally GFP-tagged, were generated and overexpressed in HEK293T cells. We note that overexpression was necessary to detect LST2 in HEK293T cells. Phospho-deficient LST2-S670A and TOS-deficient LST2-F401A were consistently less expressed compared to LST2-WT and phospho-mimetic LST2-S670E (Fig. 2 *A* and *B*), suggesting that S670 phosphorylation enhances LST2 stability. The possibility that LST2 phosphorylation promotes stability was supported by the observation that INK-128 treatment reduced LST2-WT expression (Fig. 2*C*). Unexpectedly, LST2-S670E expression was also INK-128 sensitive, which may be explained by other mTORC1 phosphorylation sites in LST2 (*SI Appendix*, Fig. S1 *G* and *H* and Dataset S1). To investigate whether LST2 is degraded by the proteosome, cells were treated with the proteasome inhibitor MG132. MG132 treatment led to increased LST2 levels in all cases (Fig. 2*C*). Thus, mTORC1-dependent LST2 phosphorylation may prevent proteasomal degradation of LST2.

To confirm that phosphorylation indeed controls LST2 stability, we measured LST2’s half-life in cells treated with the translation inhibitor cycloheximide (CHX). A cycloheximide chase allows the determination of LST2 degradation kinetics in the absence of confounding de novo LST2 synthesis. In normal growth conditions, LST2-WT had a half-life of ~24 h. Upon mTORC1 inhibition (INK-128 treatment) LST2-WT half-life was shortened to ~4 h (Fig. 2 *D* and *E*). TOS-deficient LST2-F401A and phospho-deficient LST2-S670A, in normal growth conditions, displayed short half-lives comparable to INK-128 treated LST2-WT (Fig. 2 *F*, *G*, and *I*). In contrast, phospho-mimetic LST2-S670E was stable, like LST2-WT in normal growth conditions (Fig. 2 *H* and *I*). Thus, mTORC1 phosphorylation enhances LST2 stability by preventing its proteasomal degradation.

### Phosphorylation at S670 Is Required for LST2 Ubiquitination.

**Fig. 3.**
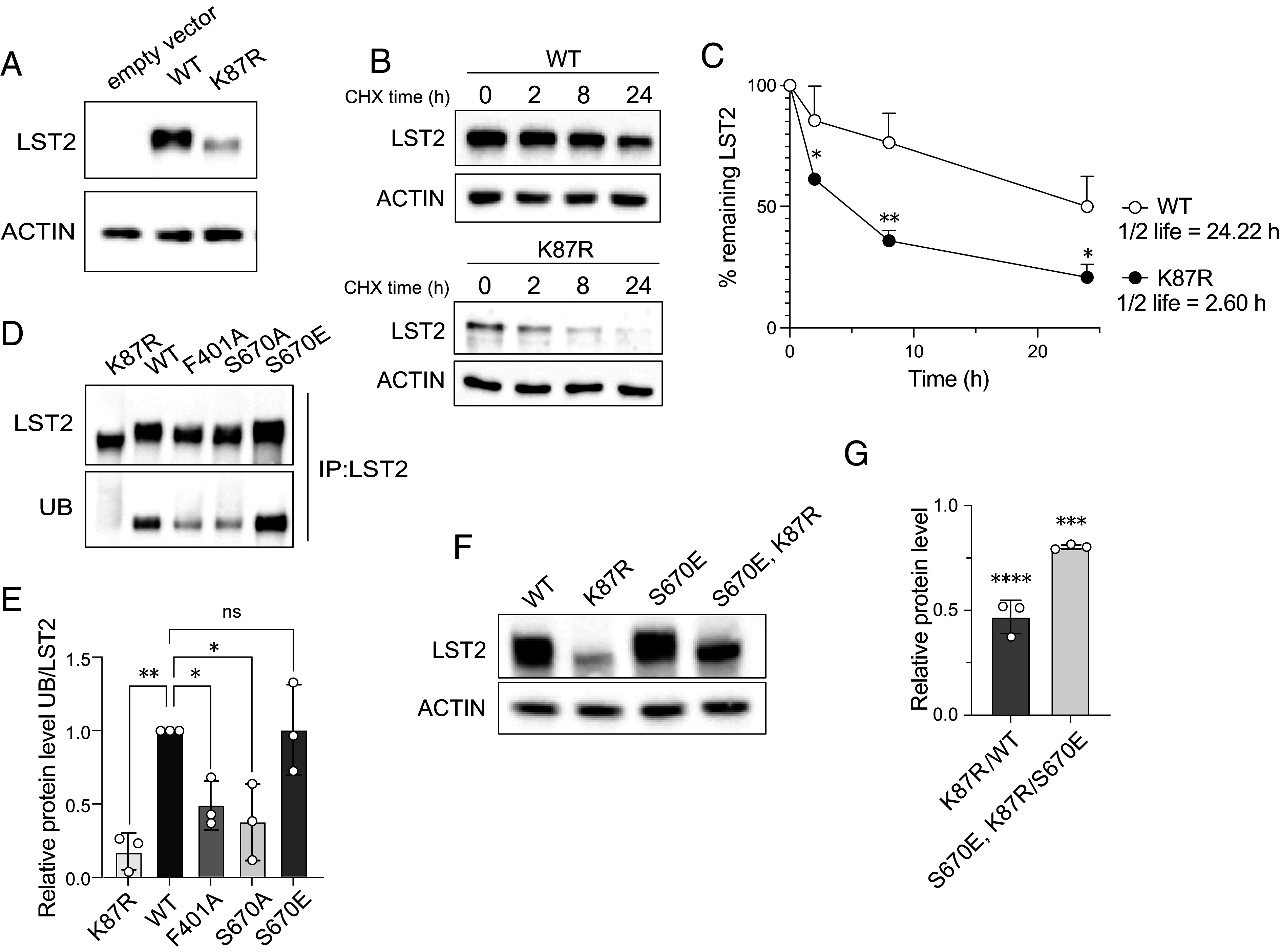
Phosphorylation on S670 is required for LST2 ubiquitination. (*A*) Immunoblots upon empty vector and LST2-WT and LST2-K87R overexpression in HEK293T cells. Media replenished 4 h before cell collection. ACTIN serves as a loading control. (*B*) Immunoblots upon LST2-WT and LST2-K87R overexpression in HEK293T cells treated with 100 µg/mL cycloheximide (CHX) for the indicated times. ACTIN serves as a loading control. (*C*) Quantification of B. Nonlinear fit. Inhibitor vs. response (three parameters), N = 3. Multiple unpaired t test, N = 3, *P < 0.05, **P < 0.01. Quantification of WT is as in Fig. 2*E*. Statistical difference is shown in comparison to WT in the illustrated time points. (*D*) HMF tagged LST2-WT, LST2-K87R, LST2-F401A, LST2-S670A, and LST2-S670E overexpressed in HEK293T cells. LST2 immunoprecipitated with flag beads. Immunoblot of equally loaded LST2. Input in *SI Appendix,* Fig S4*A*. (*E*) Quantification of D. One-way ANOVA, N = 3, *P < 0.05, **P < 0.01. (*F*) Immunoblots upon LST2-WT, LST2-K87R, LST2-S670E, LST2-S670E-K87R overexpression in HEK293T cells. Media replenished 4 h before cell collection. ACTIN serves as a loading control. (*G*) Quantification of F. LST2 levels were first normalized to ACTIN then ratio made as indicated. One-way ANOVA, N = 3, ***P < 0.001, ****P < 0.0001.

LST2 was previously reported to be monoubiquitinated on K87 (11). We generated a ubiquitin-deficient LST2-K87R mutant to investigate a potential link between LST2-K87 monoubiquitination and LST2 stability. Intriguingly, similar to TOS-deficient LST2-F401A and phospho-deficient LST2-S670A, LST2-K87R displayed lower expression levels and a reduced half-life compared to the WT (Fig. 3 *A*–*C* and Fig. 2*I*). Thus, monoubiquitination on K87 appears to promote LST2 stability, suggesting that mTORC1-mediated LST2 phosphorylation may prime monoubiquitination on K87 or vice versa. To test this, LST2-WT, -K87R, -F401A, -S670A, and -S670E were immunoprecipitated and probed with an anti-ubiquitin antibody. Loading equal total amounts of LST2, LST2-S670A, and -F401A were less ubiquitinated compared to WT and S670E (Fig. 3 *D* and *E* and *SI Appendix,* Fig. S4*A*). Moreover, the combination of S670E and K87R in *cis* reduced LST2 protein levels (Fig. 3 *F* and *G*). Thus, mTORC1-mediated LST2 phosphorylation promotes ubiquitination on K87 and ultimately LST2 stability.

### LST2 S670 Phosphorylation Promotes Reticular Distribution of LST2.

**Fig. 4.**
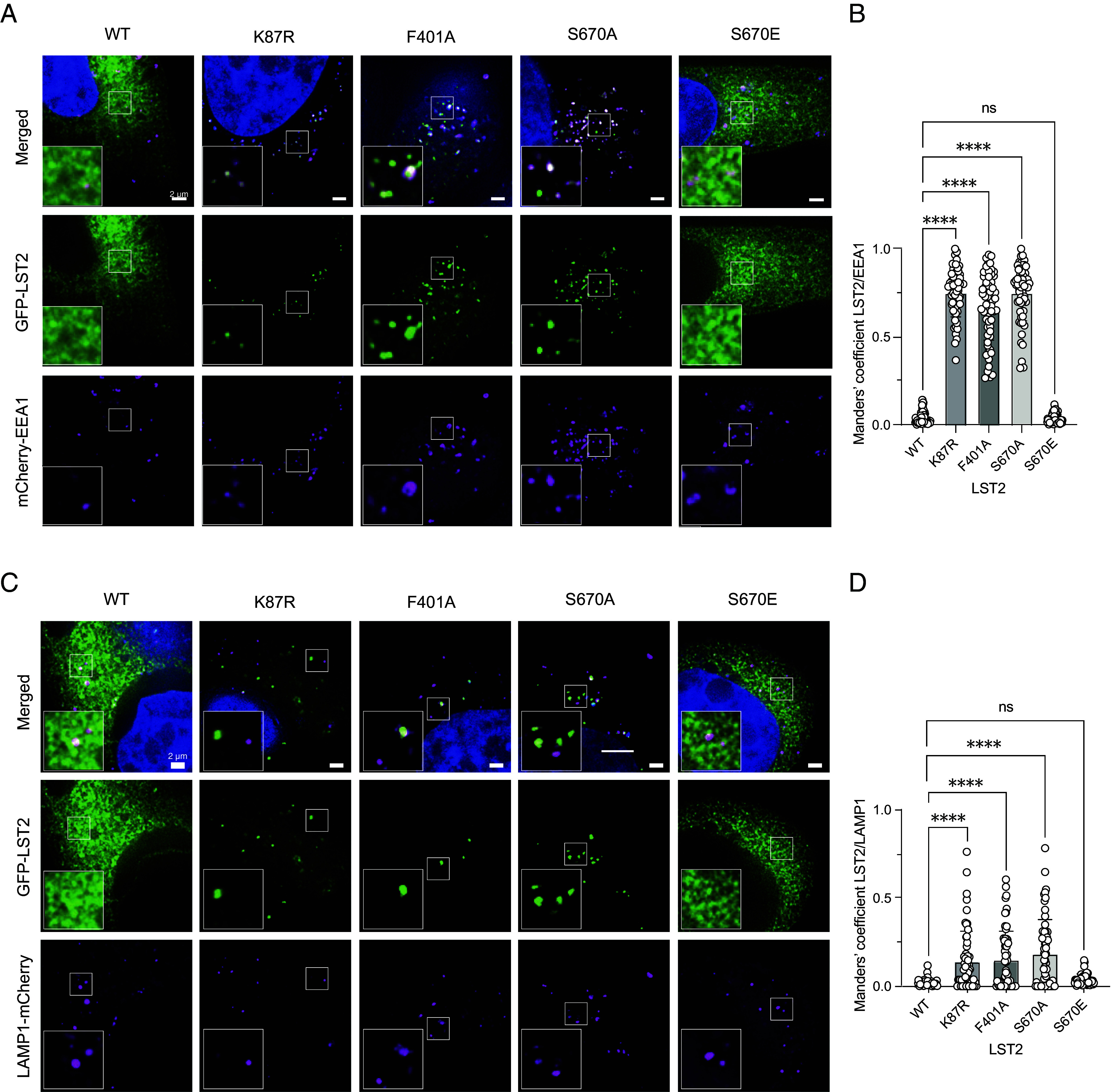
LST2 S670 phosphorylation promotes reticular distribution of LST2. (*A*) HeLa cells overexpressing mCherry tagged EEA1 in combination with GFP tagged LST2-WT, LST2-K87R, LST2-F401A, LST2-S670A, or LST2-S670E. Cells fixed in 4% PFA. In blue, DAPI staining. 2 µm bar scale. (*B*) Quantification of A. Manders’ coefficient LST2/EEA1, One-way ANOVA, N = 60, ****P < 0.0001. (*C*) HeLa cells overexpressing mCherry tagged LAMP1 in combination with GFP tagged LST2-WT, LST2-K87R, LST2-F401A, LST2-S670A, or LST2-S670E. Cells fixed in 4% PFA. In blue, DAPI staining. 2 µm bar scale. (*D*) Quantification of C. Manders’ coefficient LST2/LAMP1, One-way ANOVA, N = 60, ****P < 0.0001.

As previously reported, LST2 monoubiquitination prevents its localization to the endosomal surface. In particular, Mosesson et al. (11) showed that the LST2-K87R is endosomal, whereas LST2-WT displays a “reticular-like” distribution. Given that S670 phosphorylation promotes K87 monoubiquitination, we examined whether LST2 localization is regulated by S670 phosphorylation. TOS-deficient LST2-F401A and phospho-deficient LST2-S670A colocalized with the endosomal marker EEA1 (early endosome antigen 1), phenocopying LST2-K87R. In contrast, LST2-WT and LST2-S670E exhibited a broad reticular distribution as described previously (Fig. 4 *A* and *B* and *SI Appendix*, Fig. S5 *A* and *B*) (11). LST2-K87R, -F401A, and -S670A also weakly colocalize with the lysosomal marker LAMP1 (lysosomal associated membrane protein 1) (Fig. 4 *C* and *D* and *SI Appendix,* Fig. S5*C*). We note that LST2-WT and -S670E are broadly distributed, including to endosomes, lysosomes, and unidentified structures (Fig. 4 *A* and *C* and *SI Appendix*, Fig. S5*A*). Furthermore, the punctate endosomal fluorescence of LST2-K87R, -F401A, and -S670A is significantly weaker than the broad reticular fluorescence of LST2-WT and LST2-S670E, consistent with our previous observation that LST2-K87R, -F401A, and -S670A are unstable and thus less abundant. These data indicate that phosphorylation of S670, as previously reported for monoubiquitination of K87 (11), induces a reticular distribution of LST2.

### LST2 Is a Negative Regulator of EGFR.

**Fig. 5.**
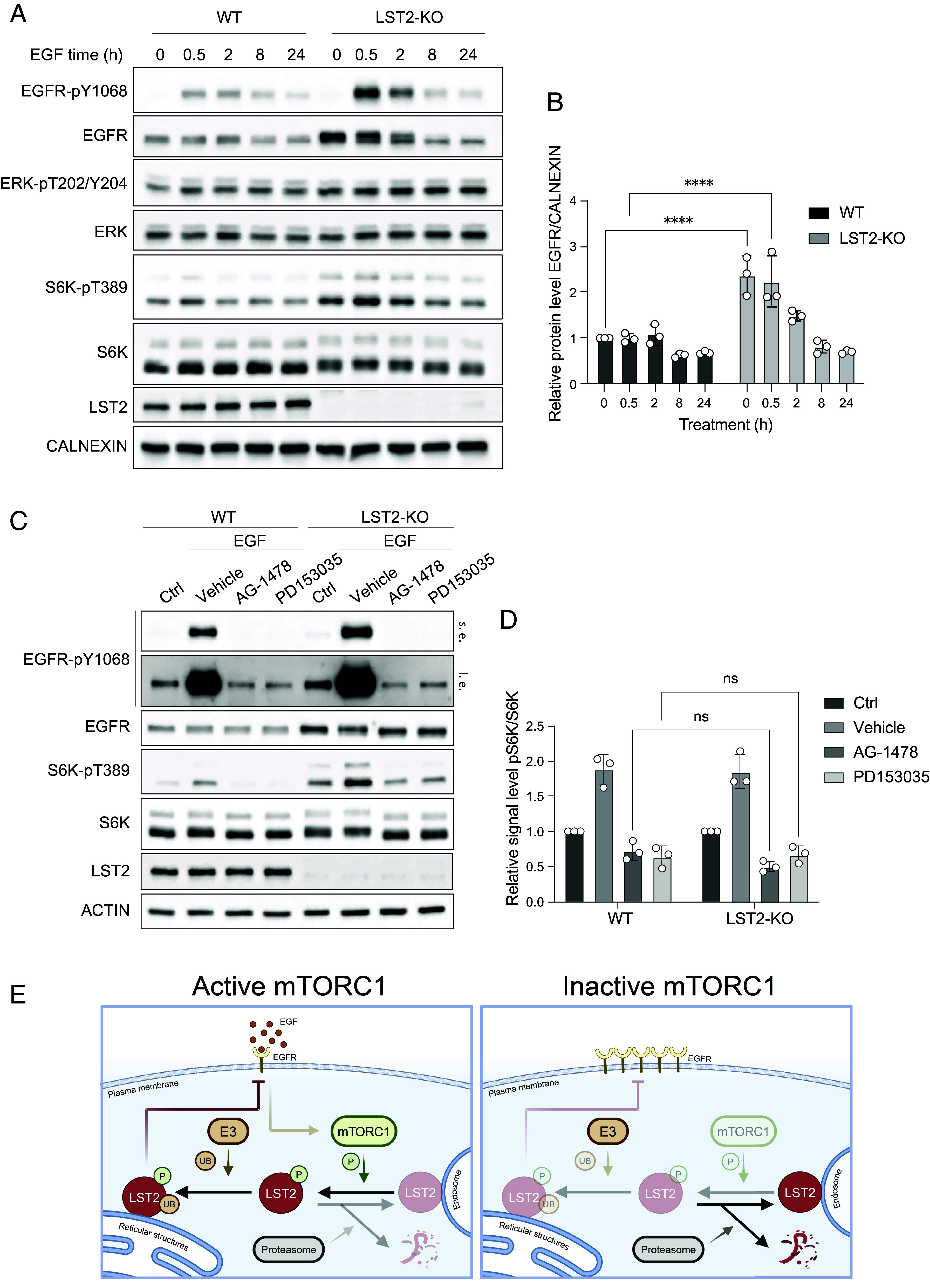
LST2 is a negative regulator of EGFR. (*A*) Immunoblot of WT and LST2-KO MDA-MB-231 cells. Cells serum-starved for 3 h and 100 ng/mL of EGF provided for the indicated time. CALNEXIN serves as a loading control. (*B*) Quantification of A. Two-way ANOVA, N = 3, ****P < 0.0001. (*C*) Immunoblot of WT and LST2-KO MDA-MB-231 cells. Cells were serum-starved for 3 h (Ctrl) and then stimulated for 30 min with 100 ng/mL of EGF. 20 µM AG-1478 or 10 µM PD153035 or 1:500 of DMSO (Vehicle) were provided 1 h before EGF as indicated. ACTIN serves as a loading control. (*D*) Quantification of C. Two-way ANOVA, N = 3. ns, no significance. (*E*) Schematic model of LST2 regulation by mTORC1. This figure has been generated with BioRender.com.

LST2 is proposed to be a negative regulator of EGFR (11, 16). EGFR is upstream of mTORC1 which, as indicated above, is upstream of LST2, suggesting that LST2 is part of a negative feedback loop in mTORC1 signaling. Is LST2 indeed part of a negative feedback loop between mTORC1 and EGFR? Because overexpression of recombinant LST2 could potentially perturb such a feedback mechanism, we sought a cell line that has a high level of endogenous LST2. By mining different expression datasets, we identified MDA-MB-231 breast cancer cells as one of the few cell lines suitably expressing LST2 (21, 22). We knocked out LST2 in MDA-MB-231 cells and examined EGFR protein levels upon serum starvation and restimulation with EGF. The levels of EGFR were increased in LST2-KO cells compared to WT cells (Fig. 5 *A* and *B*), validating the previous suggestion that LST2 negatively regulates EGFR (11, 16). Importantly, LST2-deficient cells also showed increased mTORC1 activity as indicated by increased S6K phosphorylation (Fig. 5*A*). Furthermore, inhibiting EGFR with two EGFR-specific inhibitors, AG-1478 and PD153035, reduced S6K phosphorylation to pre-stimulated levels (Fig. 5 *C* and *D*). These findings suggest that LST2 is part of a negative feedback loop from mTORC1 to EGFR.

LST2 has been linked to insulin receptor degradation, raising the possibility that LST2 is a general receptor tyrosine kinase modulator rather than an EGFR-specific regulator (14). Total levels of FGFR (fibroblast growth factor receptor), IGF1R (insulin-like growth factor 1 receptor), VEGFR2 (vascular endothelial growth factor receptor 2), and the insulin receptor β were analyzed in control and LST2-KO cells (*SI Appendix,* Fig. S6*A*). We observed that FGFR and IGF1R levels were increased and, unexpectedly, VEGFR2 and insulin receptor β levels were reduced in the absence of LST2 (*SI Appendix,* Fig. S6*A*). Thus, LST2 appears to modulate several receptor tyrosine kinases, negatively and positively depending on the receptor.

## Discussion

We show that LST2 is a mTORC1 substrate. RAPTOR binds the LST2 TOS sequence to present LST2 to the kinase catalytic site in mTOR for phosphorylation of LST2 S670. mTORC1-dependent phosphorylation at S670 leads to monoubiquitination and stabilization of LST2. LST2 in turn inhibits EGFR, generating a negative feedback loop from mTORC1 to its upstream activator EGFR (Fig. 5*E*).

LST2 was characterized previously as a negative regulator of EGFR (11, 16). Our results confirm and extend this previous finding by describing a role of LST2 in an mTORC1 negative feedback loop. EGFR activates mTORC1 through the PI3K–AKT signaling axis and mTORC1 in turn phosphorylates and stabilizes LST2 which downregulates EGFR. It is interesting to note that LST2 is not the only mTORC1 substrate that negatively feeds back on signaling upstream of mTORC1. Both S6K and GRB10 (growth factor bound-receptor protein 10) regulate stability of IRS1 (insulin receptor substrate 1), a component of the insulin receptor signaling pathway, to blunt PI3K–AKT signaling (23–28). Thus, mTORC1 appears to have different feedback mechanisms to control distinct upstream receptor tyrosine kinases (RTKs).

EGFR is not the only RTK regulated by LST2. We show that FGFR and IGF1R also accumulate in LST2-deficient cells, albeit to a lesser extent than EGFR. Furthermore, as recently reported (14), LST2 appears to mediate the degradation of activated insulin receptor. Surprisingly, we find that LST2 appears to increase insulin receptor β and VEGFR2 levels. LST2 has been proposed to regulate EGFR and insulin receptor levels via modulation of the endosomal pathway (11, 14). Exactly how LST2 discriminates between different receptors to yield different outcomes remains to be determined.

LST2 contains a FYVE domain. It was previously shown that this domain alone colocalizes with early endosomes (11). It is therefore surprising that the full-length LST2 protein exhibits a broad reticular distribution. The FYVE domain in LST2 could be sterically hindered by another domain within LST2 or by an interacting protein, upon phosphorylation and ubiquitination of LST2. Although we are unable to distinguish between cis or trans regulation, our results suggest a model where LST2 distribution changes upon phosphorylation or monoubiquitination. Given the distribution of LST2 in reticular-like structures and the increase in total EGFR levels in LST2-deficient cells, it would be informative to investigate whether LST2 acts on EGFR translation or processing in the ER.

LST2 is one of eight mTORC1 substrates with a validated TOS motif. The well-studied mTORC1 substrate 4E-BP1 also contains, in addition to a TOS sequence, a RAIP motif which synergistically strengthens the interaction of 4E-BP1 with RAPTOR (8, 19, 29). To investigate the possibility that mTORC1 binds multiple sequences in LST2, we solved the structure of mTORC1 with bound, full-length LST2. Our cryo-EM data suggest that mTORC1 recognizes LST2 exclusively through the TOS sequence and that RAPTOR binds the LST2 and 4E-BP1 TOS sequences in a highly similar manner. Superimposition of the two TOS peptides in the RAPTOR binding pocket shows that the first phenylalanine of the TOS motif lies in the same position in both structures (*SI Appendix*, Fig. S1*D*) (9). This phenylalanine has been found to be conserved in all TOS motifs reported to date (3). Our peptide-based binding analysis demonstrates that RAPTOR binds to the LST2 TOS motif with a sub-micromolar affinity. Binding of the full-length protein to RAPTOR revealed that substitution of the first phenylalanine in the TOS motif with an alanine (F401A) is sufficient to reduce the RAPTOR–LST2 interaction approximately 9-fold. Thus, the consensus phenylalanine in the LST2 TOS motif is critical for binding to RAPTOR, as also demonstrated previously for 4E-BP1 (19).

mTORC1 phosphorylates LST2 both in vitro and in vivo. Mass spectrometry reveals S670 as the main phosphorylated site, although we cannot exclude S334 and S368 as possible additional phosphorylated sites. Moreover, the stability of the S670E mutant remains INK-128 sensitive, indicating that S670 may not be the only site on LST2 phosphorylated by mTORC1. Further studies are required to elucidate the role of additional phosphorylation sites in LST2.

## Methods

Details are provided in *SI Appendix**, Material and Methods* including cell culture materials and growth conditions, immunoprecipitation-mass spectrometry and in vitro kinase assay, microscopy, fluorescence anisotropy, protein expression and purification, Cryo-EM data collection, and structure determination.

## Supplementary Material

Appendix 01 (PDF)

Dataset S01 (XLSX)

## Data Availability

Cryo-EM reconstructions and model coordinates are deposited to the EMDB and PDB for mTORC1:LST2 [map 1, *SI Appendix*, Fig. S2, EMDB ID:EMD-50184 (30), EMD-50253 (31), EMD-50254 (32), EMD-50255 (33), PDB ID:9F45 (34)], the corresponding local refinement focused on RAPTOR:LST2 [map 3, *SI Appendix*, Fig. S2, EMDB ID:EMD-50182 (35), PDB ID:9F43 (36)], and mTORC1:LST2 TOS peptide [map 4, *SI Appendix*, Fig. S3, EMDB ID:EMD-50183 (37), PDB ID:9F44 (38)], and the corresponding local refinement focused on RAPTOR:LST2 TOS peptide [map 6, *SI Appendix*, Fig. S3, EMDB ID:EMD-50181 (39), PDB ID:9F42 (40)]. The following LST2 plasmids are available at Addgene: pCMV-MLG-EGFP-LST2 (#220785) (41), pCMV-MLG-HMF-LST2 (#220786) (42), and pAceBAC2-HMF-LST2 (#220787) (43). The LST2-KO MDA-MB-231 cell line is available upon request.
