# Supplementary material for: mTORC1 phosphorylates and stabilizes LST2 to negatively regulate EGFR
Source: Proc Natl Acad Sci U S A. 2024 Aug 14;121(34):e2405959121. doi: 10.1073/pnas.2405959121 (PMC11348030; doi:10.1073/pnas.2405959121)
Supplement: Supplementary file 1 — Appendix 01 (PDF) [file pnas.2405959121.sapp.pdf]

PNAS

Supporting Information for

mTORC1 phosphorylates and stabilizes LST2 to negatively regulate EGFR

Stefania Battaglioni[1,2], Louise-Marie Craigie[1,2], Sofia Filippini[1], Timm Maier[1,*], and Michael N. Hall[1,*]

[1]Biozentrum, University of Basel
Spitalstrasse 41
4056 Basel, Switzerland

[2]Equal contribution

*Corresponding authors: m.hall@unibas.ch, timm.maier@unibas.ch

This PDF file includes:

Materials and Methods
Figures S1 to S6
Table S1

Other supporting materials for this manuscript include the following:

Dataset S1

Materials and Methods

Plasmids. Human RAPTOR was cloned into pAceBAC2 (Geneva Biotech, Geneva, Switzerland) with an N-terminal His10-myc(EQKLISEEDL)-flag(DYKDDDDK)-tag or strep-tag and Tobacco etch virus (TEV) protease cleavage site. Codon optimized DNA coding for human LST2 (Uniprot: Q9HCC9) was synthesized by Genscript. LST2 was cloned with an N-terminal His10-myc-flag-tag or EGFP-tag and TEV protease cleavage site into pCMV-MLG or pAceBAC2 (Geneva Biotech, Geneva, Switzerland), for mammalian or insect cell expression, respectively. The following LST2 plasmids were produced and are available at Addgene: pCMV-MLG-EGFP-LST2 (#220785), pCMV-MLG-HMF-LST2 (#220786) and pAceBAC2-HMF-LST2 (#220787).

Protein expression and purification. Baculovirus was generated according to (31) and Sf21 cells (94-003F, Expression Systems) were grown in HyClone SFM4Insect cell media (SH30912, GE Life Sciences). Human mTORC1 was expressed in Sf21 cells and 72 hours post infection, cells were collected by centrifugation at 1200 g for 15 minutes and stored at -80°C. The cell pellet was lysed in 50 mM Bicine-KOH pH 8.0, 200 mM NaCl and 5 mM $MgCl_2$ with a dounce homogenizer and lysate was cleared by ultracentrifugation. The cleared lysate was incubated with 5 ml anti-DYKDDDDK G1 affinity resin (Genscript) for 1 hour at 4°C. Resin was transferred to a 50 ml gravity flow column (Bio-Rad) and washed four times with 200 ml lysis buffer before incubation for one hour with 50 mM Bicine-KOH pH 8.0, 150 mM NaCl, 5 mM $MgCl_2$ and 0.6 mg/ml synthetic DYKDDDDK peptide (Genscript). Two additional elution steps were performed with 0.1 mg/ml synthetic DYKDDDDK peptide for 5 minutes and the eluates were combined. Eluted protein was concentrated using a centrifugal concentrator with regenerated cellulose membrane (UFC910024, Amicon) with 100,000 Da molecular mass cut-off. Concentrated protein was purified by size exclusion chromatography using a custom Superose 6 increase 10/600 GL gel filtration column equilibrated with 20 mM Bicine-KOH (pH 8.0), 180 mM NaCl, 5% (v/v) glycerol and 2 mM TCEP. Purified mTORC1 was concentrated and stored at -80°C.

RAPTOR with an N-terminal His10-myc-flag-tag was expressed in Sf21 cells using the same baculovirus expression system as for mTORC1. 72 hours post infection, cells were collected by centrifugation at 1200g for 15 minutes and stored at -80°C. The cell pellet was lysed in 50 mM HEPES-KOH pH 8.0, 150 mM NaCl and 1 mM TCEP with a dounce homogenizer and lysate was cleared by ultracentrifugation. The cleared lysate was filtered using a 0.8 µm YETI syringe filter (8833Y-PES-8, Infochroma), loaded onto a His Trap High Performance column (17524801, Cytiva) and washed with 10 column volumes (CV) of lysis buffer containing 80 mM imidazole. Protein was eluted using a linear gradient (10 CV) of lysis buffer to 200 mM final imidazole concentration. The tag was cleaved by incubating with TEV protease for 2 hours at 4°C. Protein was concentrated using a centrifugal concentrator with regenerated cellulose

membrane (UFC905024, Amicon) with 50,000 Da molecular mass cut-off. Concentrated protein was purified by size exclusion chromatography using a HiLoad 16/600 Superdex 200 pg gel filtration column equilibrated with 20 mM HEPES-KOH pH 8.0, 150 mM NaCl, 5% (v/v) glycerol and 5 mM TCEP. RAPTOR containing fractions were concentrated and stored at -80°C.

RAPTOR with an N-terminal strep-tag was expressed in Sf21 cells using the baculovirus expression system as for mTORC1. The cell lysate was processed as above. The lysate was loaded onto a 5 ml column packed with Strep-Tactin XT 4Flow resin (IBA Lifesciences) and washed with 15 CV of lysis buffer. Protein was eluted using 15 CV of lysis buffer containing 50 mM biotin. Protein was concentrated using a centrifugal concentrator with regenerated cellulose membrane (UFC905024, Amicon) with 50,000 Da molecular mass cut-off. Concentrated protein was dialysed against 20 mM HEPES-KOH pH 8.0, 150 mM NaCl, 5% (v/v) glycerol and 5 mM TCEP and stored at -80°C.

For expression of LST2-WT and -F401A with an N-terminal His10-myc-flag-tag, Sf21 cells were infected with baculovirus. 72 hours post infection, cells were collected by centrifugation at 1200g for 15 minutes before being stored at -80°C. The cell pellet was lysed in 20 mM HEPES-KOH pH 7.4, 200 mM NaCl, 1 mM TCEP with a dounce homogenizer and lysate was cleared by ultracentrifugation. The cleared lysate was filtered using a 0.8 µm YETI syringe filter (8833Y-PES-8, Infochroma), loaded onto a 5 ml HisTrap FF (17-5255-01, GE Healthcare) and washed with four CV of lysis buffer containing 40 mM imidazole. Protein was eluted using four CV of lysis buffer containing 200 mM imidazole. The tag was cleaved by incubating with TEV protease overnight at 4°C, followed by an orthogonal Ni-column (5ml, HisTrap FF, 17-5255-01, GE Healthcare). Protein was concentrated using a centrifugal concentrator with regenerated cellulose membrane (UFC905024, Amicon) with 50,000 Da molecular mass cutoff. Concentrated protein was purified by size exclusion chromatography using a HiLoad 16/600 Superdex 200 pg gel filtration column equilibrated with 20 mM HEPES-KOH pH 8.0, 200 mM NaCl and 2 mM TCEP. LST2 containing fractions were concentrated and stored at -80°C.

Fluorescence anisotropy binding experiments. FITC-Ahx-labelled LST2 peptides synthesized by Genscript were dissolved in DMSO and diluted in 50 mM HEPES-KOH pH 8.0, 150 mM NaCl, 5 mM TCEP and 5% (v/v) glycerol to 0.5 µM. Fluorescence anisotropy was measured with varying RAPTOR concentrations from 50 nM to 25 µM and peptide concentration was fixed at 50 nM. For the competition experiments, the concentrations of FITC-labeled TOS-peptides and RAPTOR were fixed to 50 nM and 1 µM, respectively. Fluorescence anisotropy was measured with varying concentrations of unlabelled peptides (approximately 1 µM to 200 µM). Measurements were taken using a BioTek Synergy H1M reader containing filters for excitation (485 nm) and emission (535 nm). Fluorescence anisotropy data was collected in three independently pipetted experiments.

***In vitro* co-immunoprecipitation.** Purified RAPTOR with a strep tag was incubated with purified LST2 and 'MagStrep 'type3' XT Beads' Magnetic Beads (2-4090-010, IBA lifesciences) for 2 hours at 4°C. Beads were washed four times with 250 μl lysis buffer before being incubated with lysis buffer supplemented with 50 mM Biotin (2-1016-002, IBA lifesciences) for 60 minutes at 4°C. Samples were analysed by SDS-PAGE and transferred to 0.2 μM nitrocellulose membranes using the Trans-blot Turbo Transfer System (1704150, Bio-Rad). Signal was quantified using the LI-COR Fc system (LI-COR Biosciences) with anti-RAPTOR (1:1000, RRID:AB_2130791) and anti-LST2 (1:1000, RRID:AB_10697244) antibodies. Antibodies were diluted in an equal mix of TBST and LI-COR intercept (TBS) blocking buffer (927-600, LI-COR).

Cryo-EM sample preparation and data collection. Freshly thawed mTORC1, LST2-K87R and ATPγS aliquots were mixed in a 1:4:2000 molar ratio and diluted in 10 mM Bicine-KOH pH 8.0, 20 mM NaCl, 2 mM $MgCl_2$, 0.5 mM TCEP and 1% (v/v) glycerol before grid preparation. 4 μl of sample was applied to a Quantifoil R2/1 holey carbon gold grid (Quantifoil Micro Tools), which was mounted in a Vitrobot (Thermo Fisher Scientific) where the chamber was at 8 °C and 95% humidity. The grid was blotted with a 3s blot time and plunge-frozen in liquid ethane. Data was collected using a Titan Krios (Thermo Fisher Scientific FEI) transmission electron microscope with a K2 Summit direct electron detector (Gatan) using SerialEM (RRID:SCR_017293) in counting mode. Six exposures were collected per hole, 64 frames were collected with a pixel size of 1.31 Å/pixel and an electron dose of 43 electrons/$Å^2$.

Freshly thawed mTORC1 and LST2 synthetic peptide (Genscript) were mixed in a 1:100 molar ratio and diluted in 10 mM Bicine-KOH pH 8.0, 40 mM NaCl, 2 mM $MgCl_2$, 0.5 mM TCEP and 1% (v/v) glycerol to 1 μM mTORC1 concentration before grid preparation. Grids were plunge frozen as for the mTORC1 with LST2 sample. Five exposures were collected per hole, 40 frames were collected with a pixel size of 1.31 Å/pixel and an electron dose of 48.43 electrons/$Å^2$.

Cryo-EM data processing. The mTORC1-LST2 dataset consisted of 4719 movies. Patch motion correction was performed and the contrast transfer function (CTF) parameters were determined in CryoSPARC (v4, RRID:SCR_016501) (32). After curation of movies, 4637 movies were selected for further processing and particles were picked using a template picker. 2D classification was performed in several iterations to select classes containing mTORC1 structural features and an ab-initio reconstruction was produced. After removal of duplicate particles, initial 3D homogeneous refinements using the ab-initio volume produced a reconstruction at 4.37 Å. Further homogeneous and non-uniform refinement using C2 symmetry produced a reconstruction at 4.15 Å (*SI Appendix*, Fig. S2) (33). The overall structure of mTORC1 appeared similar to other reported structures with TOS binding proteins (9). Additional density was observed only in the

TOS binding site. To prune the dataset, particles were classified using 3D classification into 10 classes. Nine classes were selected and a non-uniform refinement without symmetry produced a reconstruction at 4.02 Å (*SI Appendix*, Fig. S2). Local refinement of individual mLST8-mTOR-RAPTOR protomers produced reconstructions at 3.75 Å and 3.73 Å. A composite map (map 1 - *SI Appendix*, Fig. S2) was produced using the individually refined protomers and the overall refinement at 4.02 Å. Particles from 3D classification were symmetry expanded based on C2 symmetry of mTORC1. Using the symmetry expanded particles, a local refinement using a mask around one mTOR-mLST8-RAPTOR protomer, led to a reconstruction at 3.60 Å (map 2 - *SI Appendix*, Fig. S2). Finally, local refinements using a mask around RAPTOR produced a reconstruction at 3.49 Å resolution (map 3 - *SI Appendix*, Fig. S2).

The mTORC1 with LST2 synthetic peptide dataset consisted of 4470 movies. Motion correction and CTF fitting was applied as above. After curation, 4436 movies were selected for further processing. Particles were picked using a template picker using the ab-initio volume generated from the mTORC1-full-length LST2 dataset mentioned above. 2D classification was performed in several iterations to select classes containing mTORC1 structural features. Initial 3D homogeneous refinements with C1 symmetry, after removal of duplicate particles, produced a reconstruction at 4.02 Å. A non-uniform refinement using C2 symmetry produced a reconstruction at 3.68 Å (map 4 – *SI Appendix*, Fig. S3) (33). Particles were symmetry-expanded based on C2 symmetry of mTORC1 and a local refinement using a mask around one protomer produced a reconstruction at 3.55 Å (map 5 – *SI Appendix*, Fig. S3). The symmetry expanded particles were 3D-classified into 10 classes using a mask around RAPTOR. Nine out of ten classes, which contained density in the RAPTOR region, were merged. Finally, further local refinements using a mask around RAPTOR, global CTF refinement, and local CTF refinement where particles were split into exposure groups and defocus was estimated for each particle, resulted in a reconstruction at 3.27 Å resolution (map 6 – *SI Appendix*, Fig. S3).

Cryo-EM model building and refinement. The RAPTOR subunit of the cryo-EM structure of mTORC1 (PDB: 7PEB(34)) was used as an initial model. The LST2 TOS peptide sequence was built and adjusted manually against map 6 using COOT. The structure was real-space-refined against map 6 using phenix.real_space_refine (35, 36). The resulting model was used as a starting model for refinement against the LST2-mTORC1 dataset (map 3) using phenix.real_space_refine. For full-length LST2-mTORC1 model building, one protomer of 7PEB was used for rigid body fitting and subsequent real-space-refinement against map 2. Two mTORC1-LST2 protomers were then used for refinement against map 1 using phenix.real_space_refine. All models were validated using Phenix (36) and MolProbity (37).

Structural analysis and figure generation. Properties of protein interface between LST2 and RAPTOR was analyzed using PISA (38) (RRID:SCR_015749). All density and structure representations were generated using UCSF ChimeraX (version 1.7, RRID:SCR_015872). Surface and electrostatic calculations were also performed in ChimeraX. To analyze LST2 and 4E-BP1 peptides, model PDB: 6BCX was superimposed based on RAPTOR. Resolutions were determined using gold standard FSC curves in cryoSPARCv4. For display, maps have been smoothened in ChimeraX.

Cell culture. HeLa cell line was purchased from DMSZ (ACC 57, lot 28), HEK293T and MDA-MB-231 cell lines are from our laboratory collection. All cell lines used in the study were tested and found free of mycoplasma. All cell lines were grown in DMEM high glucose (D5671, Sigma) media supplemented with 10 % FBS (10500064, Gibco), glutamine (25030081, Gibco), 0.1 mM non-essential amino acids (11140-035, Gibco), sodium pyruvate (11360070, Gibco), and 1X penicillin-streptomycin (15140122, Thermo Fisher). Where indicated cells were treated with 200 nM INK-128 (MLN0128, Axon MedChem), 100 µg/mL of cycloheximide (C7698, Sigma), 10 µM MG132 (S2619, Selleckchem), 20 µM AG-1478 (HY-13524, MedChemExpress), 10 µM PD153035 (HY-14346, MedChemExpress) and DMSO (41640, Sigma). Cells were incubated at 37 °C with 5% CO_2.

Immunoblot analysis. Cells were washed once in ice-cold PBS then lysed in M-PER (78501, Thermo Scientific) supplemented with 1x Complete Mini Protease Inhibitor (11836153001, Roche) and 1x PhosSTOP (4906845001, Roche) on ice. Lysate was shaken at 1400 rpm for 10 min at 4 °C then debris removed by centrifugation (18000 g, 10 min at 4 °C). Protein concentration was calculated with BCA assay (23250, Thermo Scientific). Equal amounts of homogenates loaded and resolved by SDS–PAGE and transferred onto a nitrocellulose membrane (GE Healthcare). LST2 antibody was purchased from Atlas antibodies (HPA038175). From Cell Signaling Technology the following antibodies were employed: EGFR (2232), p-EGFR Tyr1068 (3777), ERK (4696), p-ERK Thr202/Tyr204 (9106), S6K (2708), p-S6K Thr389 (9234), mTOR (2972), mLST8 (3274), RAPTOR (2280), AKT (4685), p-AKT Ser473 (4060), RICTOR (2140), GAPDH (97166), FGFR (9740), VEGF2 (9698), IGF1R (3018) and insulin receptor β (3025). Anti-ACTIN antibody was purchased from Millipore (MAB1501), anti-CALNEXIN from Enzo (ADI-SPA-860), anti-Ubiquitin from SantaCruz Biotechnology (sc-8017). Secondary HRP-conjugated antibodies were purchased From Jackson immunoresearch, anti-rabbit (211-032-171) and anti-mouse (115-035-174).

EGF treatment. MDA-MB-231 LST2-KO cells were generated with CRISPR Cas9 (sc-412894, Santa Cruz Biotechnology) and single clones sequenced to confirm proper deletion. WT and

LST2-KO cells were plated in equal density and allowed to attach for 24 hours. Cells were then starved in serum-free media (DMEM high glucose media supplemented with glutamine, nonessential amino acids, sodium pyruvate, and penicillin-streptomycin) for 3 hours, then 100 ng/mL of human EGF (Sigma Aldrich, E9644) was provided and cells lysed at different time points as described above.

Cycloheximide chase. HEK293T cells were transfected in 15 cm plates with 8 µg GFP-LST2 WT plasmid and in 10 cm plates with 4 ug GFP-LST2 plasmids: either K87R, F401A, S670A or S670E. One 15 cm plate was split into eight 6 cm plates. Each 10 cm plate was split into four 6 cm plates. Fresh media was provided 1 hour before the beginning of the experiment. 0-hour time point was collected just before treatment started. Then, 100 µg/mL of cycloheximide was added to all cells and cells collected at 2, 8 and 24 hours post treatment. For INK-128 treatment either 1:1000 DMSO or 200 nM INK-128 was added together with CHX. Cells were processed for immunoblot as described.

Immunoprecipitation (IP). HEK293T cells were processed up to protein concentration determination as for immunoblot analysis (see above). Then, 1.5 mg of protein was mixed with 30 µL beads (GFP-Trap from ChromoTek or Anti-Flag M2 magnetic beads from Sigma Aldrich, F3165) in 2 mL M-PER buffer and gently rotated for 3 hours at 4°C. After washing 3 times with M-PER buffer, a final wash was performed in 20 mM Tris-HCl (pH 7.5), 150 mM NaCl. Proteins were either digested on beads for mass spectrometry analysis (see below) or eluted by addition of 2x Laemmli buffer for immunoblot.

In vitro kinase assay. *In vitro* kinase assay was performed in the following buffer: 50 mM HEPES-KOH pH 7.4, 0.5 mM EGTA, 1.25 mM DTT, 0.00125% Tween-20 and 5 mM $MnCl_2$. Each reaction contained 10 µM of radioactive [γ-32P]-ATP (2.5 µCi), 10nM of mTORC1, 400 nM substrate (dephosphorylated LST2-WT or -F401A or positive control S6K) and either 1:100 of DMSO or 10 µM of INK-128 or 25 µM Torin-1. Mastermix of reaction buffer with mTORC1 (or water in negative control) and either DMSO or INK-128 or Torin-1 were allowed to equilibrate for 5 minutes on ice. Radioactive ATP was added and the mastermix was equally divided into reaction tubes containing the substrate (reaction buffer in control). Samples were incubated with gentle agitation at 37°C for 30 min. 5x laemmli buffer was added and samples were heated for 10 min at 65°C. Immunoblot was performed as described previously and membrane was exposed overnight and radioactive signal was visualized with phosphoimager Typhoon FLA 7000. Once the radioactive signal was captured, the membrane was probed with antibodies to determine protein loading.

Microscopy. HeLa cells were transfected with GFP-LST2 plasmid, WT, K87R, F401A, S760A, S670E, and either mCherry-EEA1 (pFX-mCherry-EEA1 was a gift from Yusuke Ohba, Addgene, 174452) or LAMP1-mCherry (pLAMP1-mCherry was a gift from Amy Palmer, Addgene, 45147). Cells were plated in equal number in microscopy chamber (81816, Ibidi) and fixed in 4% PFA with addition of NucBlue™ (R37605, Invitrogen) for 10 minutes at room temperature. Cells were then washed once in PBS and kept in PBS during visualization. Cells were imaged with Widefield microscope Olympus IX71 in 60x oil-immersed objective (Plan Apo N) with extra 1.6x magnification. Images were deconvoluted with SoftWorx 4.1.2 and OMERO was used for Image Data Management. Image quantification was performed in Image J with Jacop plugin on 5 µm x 5 µm squares. 20 squares were chosen per condition and per replicate, with maximum 3 squares per cells. Experiment performed in three independent replicates.

Proteomic analysis of *in vitro* kinase assay. LST2-WT and LST2-F401A were phosphorylated *in vitro* by mTORC1 (as described above, without radioactivity) in the presence or absence of INK-128. At the end of the reaction, samples were resuspended in lysis buffer (5% SDS, 10 mM TCEP, 0.1 M TEAB), incubated for 10 min at 95°C, alkylated in 20 mM iodoacetamide for 30 min at 25°C and digested using S-Trap™ micro spin columns (Protifi) according to the manufacturer's instructions. Briefly, 12 % phosphoric acid was added to each sample (final concentration of phosphoric acid 1.2%) followed by the addition of S-trap buffer (90% methanol, 100 mM TEAB pH 7.1) in a ratio of 6:1. Samples were mixed by vortexing and loaded onto S-trap columns by centrifugation at 4000 g for 1 min followed by three washes with S-trap buffer. Digestion buffer (50 mM TEAB pH 8.0) containing sequencing-grade modified trypsin (1/25, w/w; Promega, Madison, Wisconsin) was added to the S-trap column and samples were incubated for 1 h at 47 °C. Peptides were eluted by the consecutive addition and collection by centrifugation at 4000 g for 1 min of 40 µl digestion buffer, 40 µl of 0.2% formic acid and finally 35 µl 50% acetonitrile, 0.2% formic acid. Samples were dried under vacuum and stored at -20 °C until further use.

Dried peptides were resuspended in 0.1% aqueous formic acid and subjected to LC–MS/MS analysis using a Orbitrap Fusion Lumos Mass Spectrometer fitted with an EASY-nLC 1200 (both Thermo Fisher Scientific) and a custom-made column heater set to 60°C. Peptides were resolved using a RP-HPLC column (75µm × 36cm) packed in-house with C18 resin (ReproSil-Pur C18–AQ, 1.9 µm resin; Dr. Maisch GmbH) at a flow rate of 0.2 µl/min. The following gradient was used for peptide separation: from 5% B to 12% B over 5 min to 35% B over 40 min to 50% B over 15 min to 95% B over 2 min followed by 18 min at 95% B. Buffer A was 0.1% formic acid in water and buffer B was 80% acetonitrile, 0.1% formic acid in water.

The mass spectrometer was operated in DDA mode with a cycle time of 3 seconds between master scans. Each master scan was acquired in the Orbitrap at a resolution of 240,000 FWHM (at 200 m/z) and a scan range from 375 to 1600 m/z followed by MS2 scans of the most

intense precursors in the Orbitrap at a resolution of 30,000 FWHM (at 200 m/z) with isolation width of the quadrupole set to 1.4 m/z. Maximum ion injection time was set to 50ms (MS1) and 54 ms (MS2) with an AGC target set to 1e6 and 5e4, respectively. Only peptides with charge state 2 – 5 were included in the analysis. Monoisotopic precursor selection (MIPS) was set to Peptide, and the Intensity Threshold was set to 2.5e4. Peptides were fragmented by HCD (Higher-energy collisional dissociation) with collision energy set to 35%, and one microscan was acquired for each spectrum. The dynamic exclusion duration was set to 30s.

The acquired raw-files were searched using MSFragger (v. 3.4) implemented in FragPipe (v. 17.1) against a Spodoptera frugiperda database (consisting of 26778 protein sequences downloaded from Uniprot on 20210217) spiked with the sequence of human mTOR (P42345) and LST2 (Q9HCC9) and 392 commonly observed contaminants using the “LFQ-MBR” workflow. Phosphorylation (STY) was applied as variable modification.

Proteomic analysis of immunoprecipitated LST2 from cells. HEK293T were plated in nine 15 cm plates and each plate was transfected with 3 µg of plasmid, either GFP, GFP-LST2 WT or GFP-LST2 F401A, in triplicates. After 24 hours each plate was split into 3 plates (total number of plates, 27). After 48 hours cells were washed once in PBS and serum-deprived media was provided for 18 hours. Next, cells were treated for 2 hours with either full media (fed), full media with 200 nM INK-128 (INK-128) or left in serum-deprived media (starved). Cells were collected and immunoprecipitated with GFP-trap beads as described above.

Affinity purified samples were subjected to on-bead digestion (adapted from (39)). Resin was washed three times with detergent free wash solution and collected by centrifugation. Proteins were eluted by incubation in 1.6 M urea, 100 mM ammonium bicarbonate, 5 µg/ml trypsin, pH 8 for 30 min at 27°C followed by two incubations in 1.6 M urea, 100 mM ammonium bicarbonate, 1 mM TCEP, pH 8. After each incubation, resin was collected by centrifugation and supernatant was collected and pooled. TCEP and chloroacetamide were added at a final concentration of 10 mM and 15 mM, respectively, and samples were incubated for 1 h at 37° prior to the addition of 0.5 µg trypsin and incubation for 12 h at 37°C. Tryptic digest was acidified (pH<3) using TFA and desalted using C18 reversed phase spin columns (Microspin, Harvard Apparatus) according to the manufacturer’s instructions. Peptides were dried under vacuum and stored at -20°C. LC elution grading performed as described above.

The mass spectrometer was operated in DDA mode with a total cycle time of approximately 1 s. Each MS1 scan was followed by high-collision-dissociation (HCD) of the 10 most abundant precursor ions with dynamic exclusion set to 45 seconds. MS1 scans were acquired at resolution of 70,000 FWHM (at 200 m/z), scan range set to 350 – 1600 m/z, with an AGC target of 3e6 and a maximum injection time of 100 ms. MS2 scans were acquired at a resolution of 35,000 FWHM (at 200 m/z), scan range set to 200 – 20000 m/z, with an AGC target

of 1e5 and a maximum injection time of 100 ms. Singly charged ions and ions with unassigned charge state were excluded from triggering MS2 events. The normalized collision energy was set to 27%, the mass isolation window was set to 1.4 m/z and one microscan was acquired for each spectrum.

The acquired raw-files were searched using MSFragger (v. 3.5) implemented in FragPipe (v. 18.0) against a Homo sapiens database (consisting of 20372 protein sequences downloaded from Uniprot on 2022-02-22) and 392 commonly observed contaminants using the “Default” workflow. Phosphorylation (STY) was applied as variable modification.

Quantification and statistical analysis. Statistical significance between multiple groups was determined with 2-way ANOVA. Band intensities in immunoblot blots were measured with Image J. Statistical analysis and data plotting were performed using GraphPad Prism 9 or 10 (GraphPad Software). Statistical significance was defined as $p < 0.05$. Data represent mean ± SEM. Sample numbers are indicated in each figure legend. For the fluorescence anisotropy experiments, dissociation constants were calculated using GraphPad Prism to fit the data via a one-site binding model, errors were quantified based on profile likelihood and error bars represent the 95% confidence intervals.

Figure S1

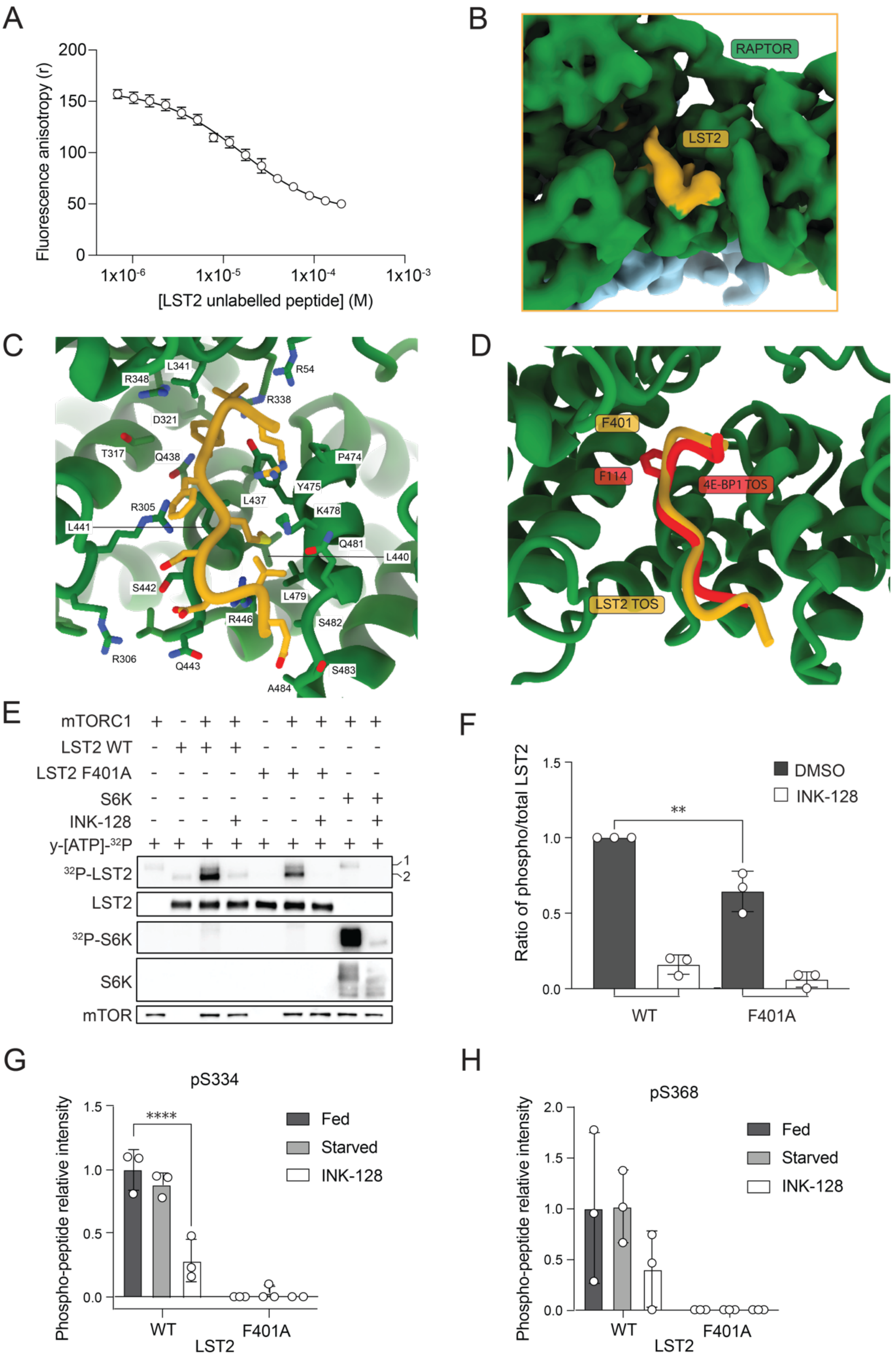
A
Fluorescence anisotropy (r)
200
150
100
50
0
1x10-6
1x10-5
1x10-4
1x10-3
[LST2 unlabelled peptide] (M)
B
RAPTOR
LST2
C
R348
L341
R54
R338
D321
T317
Q438
P474
Y475
L437
R305
K478
L441
Q481
L440
S442
L479
R446
S482
R306
Q443
S483
A484
D
F401
F114
4E-BP1 TOS
LST2 TOS
E
mTORC1
LST2 WT
LST2 F401A
S6K
INK-128
y-[ATP]-32P
32P-LST2
1
2
LST2
32P-S6K
S6K
mTOR
F
Ratio of phospho/total LST2
1.5
1.0
0.5
0.0
DMSO
INK-128
**
WT
F401A
G
pS334
Phospho-peptide relative intensity
1.5
1.0
0.5
0.0
****
Fed
Starved
INK-128
WT
F401A
LST2
H
pS368
Phospho-peptide relative intensity
2.0
1.5
1.0
0.5
0.0
Fed
Starved
INK-128
WT
F401A
LST2

Fig. S1. mTORC1 interacts with LST2 and phosphorylates LST2 at multiple sites. (A) Competitive binding of labelled (50 nM) versus unlabeled LST2 TOS peptide with RAPTOR (1μM) measured by fluorescence anisotropy, N=3. **(B)** Cryo-EM reconstruction of full-length LST2 bound to mTORC1, displaying a zoom onto the region indicated by a yellow box in Fig. 1E. A map at 3.49 Å resolution (map 3 from *SI Appendix,* Fig. S2) from a local refinement is contoured at 0.2 electron/$Å^2$, displaying the same region indicated in Fig. 1, panel E by a box, coloring corresponds to Fig. 1, panel E, F. **(C)** Interactions of the LST2 TOS peptide bound to the RAPTOR TOS-binding pocket. View is shown as in figure 1H. RAPTOR is displayed in green and residues interacting with the LST2 TOS peptide are displayed as sticks and labelled. The LST2 peptide is shown in cartoon representation in dark yellow. Side chains are colored by heteroatom with light yellow for sulfur, blue for nitrogen and red for oxygen. D404 of the TOS peptide forms a salt bridge with the neighboring R305 RAPTOR residue. V400, F401, F402, D404, D405 and E407 of the LST2 peptide form hydrogen bonds with surrounding RAPTOR residues Y475, L441, R305, R446 and S483. **(D)** Comparison of the binding pose of the TOS peptides of 4E-BP1 (PDB:6BCX) and LST2 based on a superposition of RAPTOR. The peptides are shown as tubes, the conserved TOS motif phenylalanine residue (4E-BP1-F114, LST2-F401) is shown as a stick-model. LST2 and 4E-BP1 peptides are shown in yellow and red, respectively. 4E-BP1 TOS peptide binding mode is representative of all solved TOS peptides. **(E)** Radioactive *in vitro* mTORC1 kinase assay with LST2-WT and LST2-F401A. Each reaction was carried out with 10 μM of radioactive [γ-^{32}P]-ATP (2.5 μCi). The SDS-PAGE gel was first transferred then exposed for radioactive detection and membrane probed with indicated antibodies. **(F)** Quantification of E. One-way ANOVA, N=3, ** $p < 0.01$. **(G)** Quantitative mass spectrometric analysis of LST2 phosphorylation on S334. Intensities have been normalized for WT Fed conditions. 2-way ANOVA, N=3, **** $p < 0.0001$**. (H)** Quantitative mass spectrometric analysis of LST2 phosphorylation on S368. Intensities have been normalized for WT Fed conditions.

Figure S2

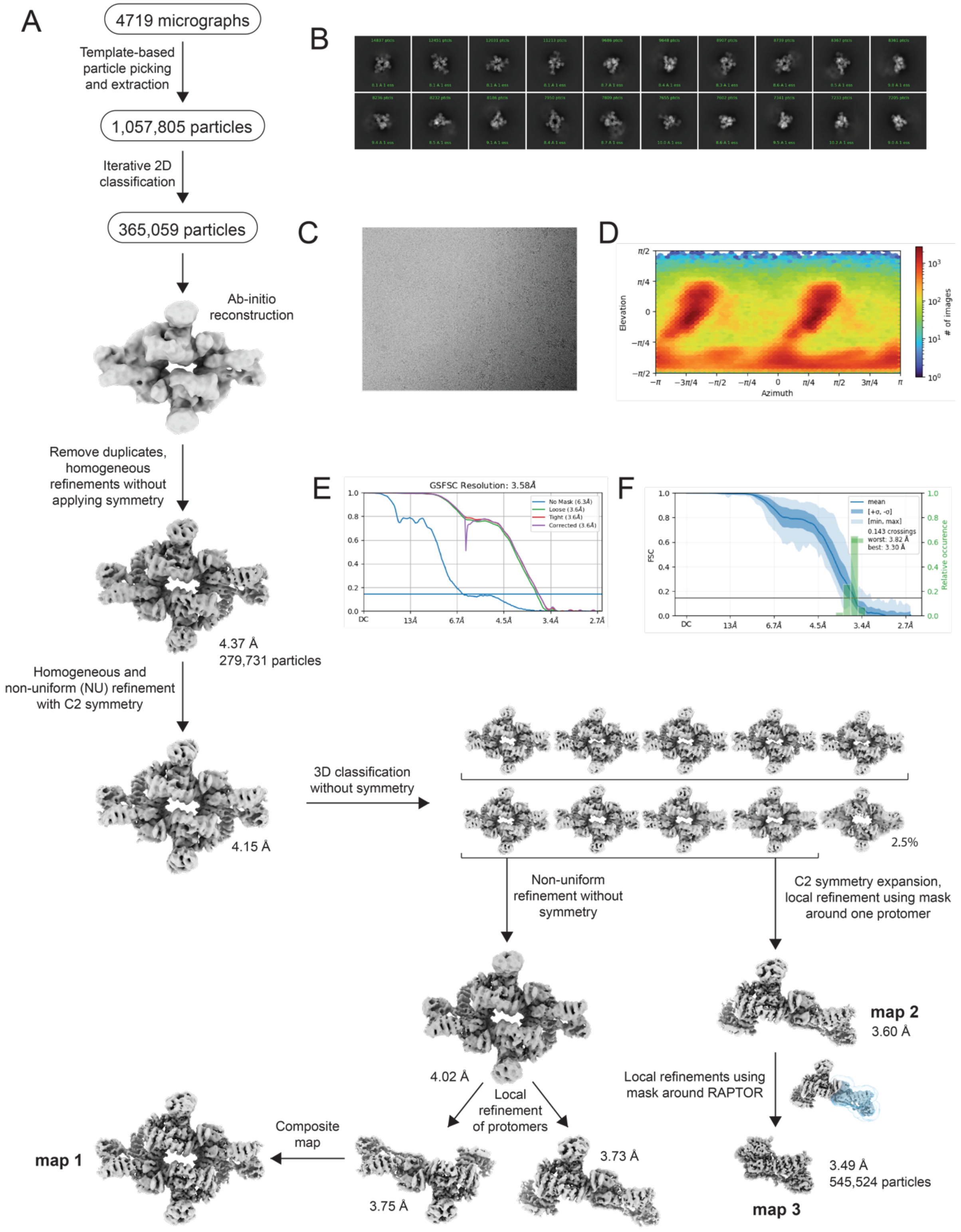
A
4719 micrographs
Template-based particle picking and extraction
1,057,805 particles
Iterative 2D classification
365,059 particles
Ab-initio reconstruction
Remove duplicates, homogeneous refinements without applying symmetry
4.37 Å
279,731 particles
Homogeneous and non-uniform (NU) refinement with C2 symmetry
4.15 Å
3D classification without symmetry
2.5%
Non-uniform refinement without symmetry
C2 symmetry expansion, local refinement using mask around one protomer
map 2
3.60 Å
4.02 Å
Local refinement of protomers
Local refinements using mask around RAPTOR
Composite map
map 1
3.75 Å
3.73 Å
3.49 Å
545,524 particles
map 3
B
C
D
Elevation
Azimuth
# of images
E
GSFSC Resolution: 3.58Å
No Mask (6.3Å)
Loose (3.6Å)
Tight (3.6Å)
Corrected (3.6Å)
F
FSC
Relative occurence
mean
0.143 crossings
worst: 3.82 Å
best: 3.30 Å

Fig. S2. Cryo-EM processing of LST2 in complex with mTORC1. (A) Schematic and simplified representation of the cryo-EM processing workflow. mTORC1-LST2 overall refinement (map 1), focused refinement on symmetry expanded mTOR-mLST8-RAPTOR protomer (map 2), and focused refinement on protomer focusing on RAPTOR (map 3) were used for modelling and illustration. **(B)** 20 most populated 2D class averages in final 2D classification iteration, indicating number of particles present in each class. **(C)** Representative micrograph of the mTORC1-LST2 dataset. **(D)** Viewing direction distribution from CryoSPARC of the focused refinement on RAPTOR (map 3). **(E)** Fourier shell correlation (FSC), after FSC-mask auto-tightening from CryoSPARC. Curves are shown for unmasked, loose, tight masks, and corrected FSC for the map 3 reconstruction. **(F)** Summary of 3072 conical FSCs from CryoSPARC for the map 3 reconstruction.

Figure S3

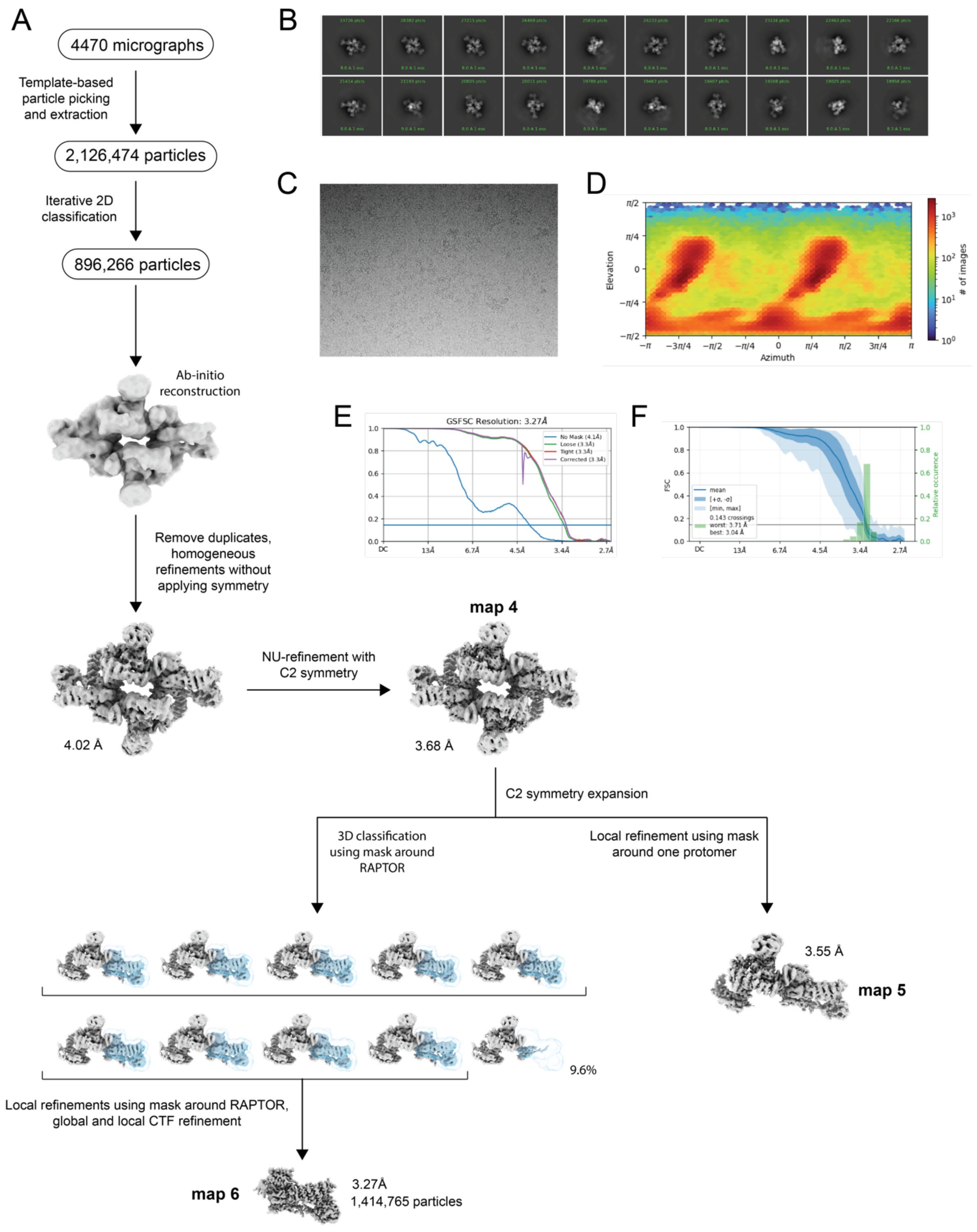

A
4470 micrographs
Template-based particle picking and extraction
2,126,474 particles
Iterative 2D classification
896,266 particles
Ab-initio reconstruction
Remove duplicates, homogeneous refinements without applying symmetry
4.02 Å
NU-refinement with C2 symmetry
map 4
3.68 Å
C2 symmetry expansion
3D classification using mask around RAPTOR
Local refinement using mask around one protomer
3.55 Å
map 5
9.6%
Local refinements using mask around RAPTOR, global and local CTF refinement
map 6
3.27Å
1,414,765 particles
B
C
D
Elevation
Azimuth
# of images
E
GSFSC Resolution: 3.27Å
No Mask (4.1Å)
Loose (3.3Å)
Tight (3.3Å)
Corrected (3.3Å)
F
FSC
mean
0.143 crossings
worst: 3.71 Å
best: 3.04 Å
Relative occurence

Fig. S3. Cryo-EM processing of LST2 TOS peptide in complex with mTORC1. (A) Schematic and simplified representation of the cryo-EM processing workflow. mTORC1-LST2 TOS peptide overall refinement (map 4), focused refinement on symmetry expanded protomer (map 5) and focused refinement on protomer focusing on RAPTOR (map 6) were used for modelling and illustration. **(B)** Top 20 2D class averages in final 2D classification iteration, indicating number of particles present in each class. **(C)** Representative micrograph of the mTORC1-LST2 TOS peptide dataset. **(D)** Viewing direction distribution from CryoSPARC of the mTORC1-peptide overall refinement (map 4). **(E)** Fourier shell correlation (FSC) after FSC-mask auto-tightening from CryoSPARC. Curves are shown for unmasked, loose, tight masks, and corrected FSC curve for the map 6 reconstruction. **(F)** Summary of 3072 conical FSCs from CryoSPARC for the map 6 reconstruction.

Figure S4

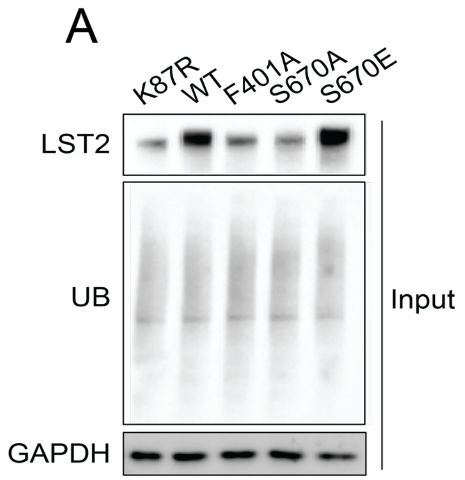

Fig. S4. Phosphorylation on S670 is required for LST2 ubiquitination. (A) Immunoblots upon HMF tagged LST2-WT, LST2-K87R, LST2-F401A, LST2-S670A and LST2-S670E overexpression in HEK293T. Input of immunoprecipitation in Fig. 3D. GAPDH serves as a loading control.

Figure S5

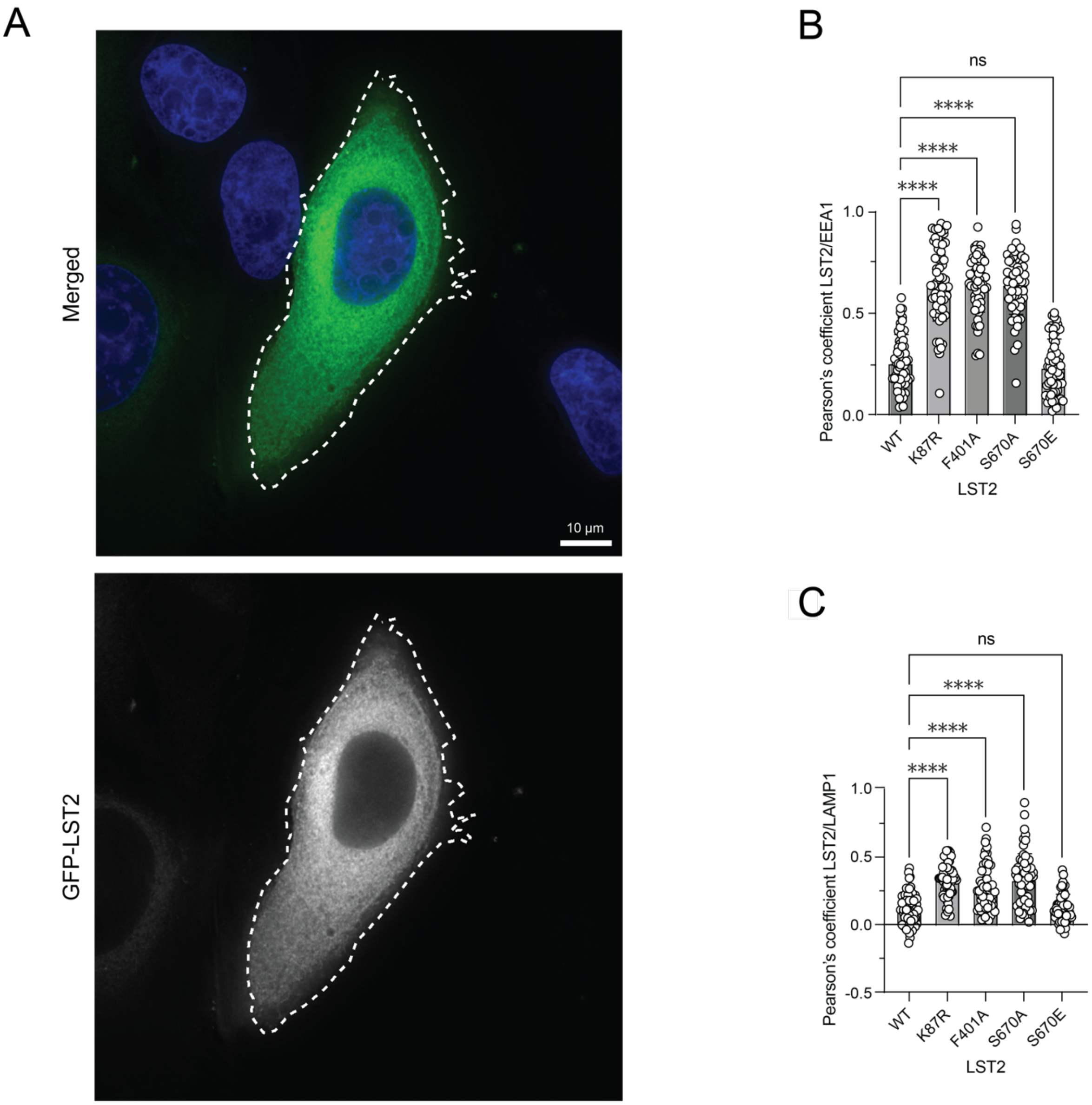

Fig. S5. LST2 has a reticular distribution. (A) HeLa cells overexpressing GFP tagged LST2-WT. Cells fixed in 4% PFA. In blue, DAPI staining. Edges of the cells are marked by dotted line. 10 µm scale bar. **(B)** Quantification of Fig. 4A. Pearson's coefficient of analysis performed in Fig. 4B. One-way ANOVA, N=60, **** $p < 0.0001$. **(C)** Quantification of Fig. 4C. Pearson's coefficient of analysis performed in Fig. 4D. One-way ANOVA, N=60, **** $p < 0.0001$.

Figure S6

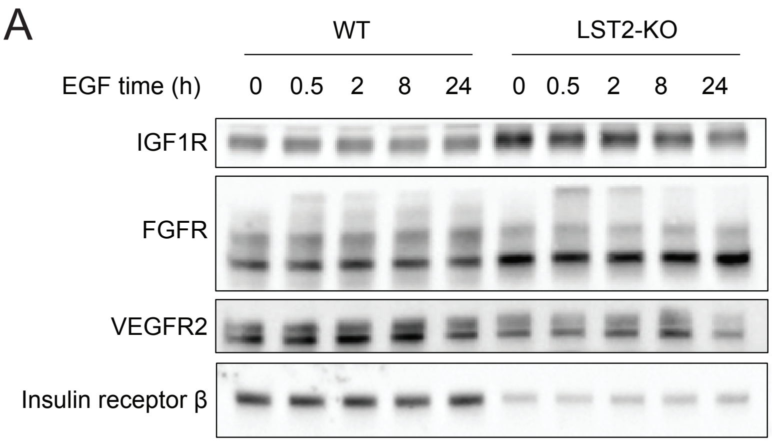

Fig. S6. Regulation of other RTKs by LST2. (A) Immunoblot of WT and LST2-KO MDA-MB-231 cells. Cells serum-starved for 3 hours and 100 ng/mL of EGF provided for the indicated time. This blot is a continuation of Figure 5A.

Table S1. Cryo-EM data collection and refinement statistics of mTORC1-LST2 complex.

mTORC1-LST2				
Dataset	mTORC1-LST2		mTORC1-LST2 TOS peptide	
Map refinement	Overall (map 1, *SI Appendix,* Fig. S2)	Focused on RAPTOR (map 3, *SI Appendix,* Fig. S2)	Overall (map 4, *SI Appendix,* Fig. S3)	Focused on RAPTOR (map 6, *SI Appendix,* Fig. S3)
**Data acquisition and processing**				
EMDB accession #	EMD-50184	EMD-50182	EMD-50183	EMD-50181
Microscope	Titan Krios			
Magnification	105			
Voltage (kV)	300			
Electron exposure ($e^-/Å^2$)	43		48.43	
Frames	64		40	
Defocus range (μM)	-1.0 to -2.5			
Pixel size (Å)	1.31			
Number of movies	4719		4470	
Symmetry imposed	C1	C1	C2	C1
Initial particles	1057805		2126474	
Final particles	272762	545524	782845	1414765
Map resolution (Å)	3.74	3.49	3.68	3.27
FSC threshold	0.143			
**Model refinement**				
PDB ID	9F45	9F43	9F44	9F42
MolProbity score	1.81	1.37	1.67	1.76
Clashscore	8.39	5.62	7.03	6.92
Rotamer Outliers (%)	2.01	0.21	1.65	1.81
R.M.S. deviations				
Bond length (Å)	0.002	0.002	0.002	0.002
Bond angle (°)	0.443	0.465	0.477	0.455
Ramachandran				
Favored (%)	97.33	97.80	97.37	96.94
Allowed (%)	2.67	2.20	2.63	3.06
Disallowed (%)	0.00	0.00	0.00	0.00

Dataset S1 (separate file). Mass spectrometry raw data collection.
